# Environmental Determinants of Chronic Disease and Medical Approaches: Recognition, Avoidance, Supportive Therapy, and Detoxification

**DOI:** 10.1155/2012/356798

**Published:** 2012-01-19

**Authors:** Margaret E. Sears, Stephen J. Genuis

**Affiliations:** ^1^Children's Hospital of Eastern Ontario Research Institute, and Senior Clinical Research Associate, Ottawa Hospital Research Institute, Ottawa, ON, Canada K1H 8L1; ^2^RR 1, Box 9012, Dunrobin, ON, Canada K0A 1T0; ^3^Faculty of Medicine, University of Alberta, Edmonton, AB, Canada T6K 4C1

## Abstract

The World Health Organization warns that chronic, noncommunicable diseases are rapidly becoming epidemic worldwide. Escalating rates of neurocognitive, metabolic, autoimmune and cardiovascular diseases cannot be ascribed only to genetics, lifestyle, and nutrition; early life and ongoing exposures, and bioaccumulated toxicants may also cause chronic disease. Contributors to ill health are summarized from multiple perspectives—biological effects of classes of toxicants, mechanisms of toxicity, and a synthesis of toxic contributors to major diseases. Healthcare practitioners have wide-ranging roles in addressing environmental factors in policy and public health and clinical practice. Public health initiatives include risk recognition and chemical assessment then exposure reduction, remediation, monitoring, and avoidance. The complex web of disease and environmental contributors is amenable to some straightforward clinical approaches addressing multiple toxicants. Widely applicable strategies include nutrition and supplements to counter toxic effects and to support metabolism; as well as exercise and sweating, and possibly medication to enhance excretion. Addressing environmental health and contributors to chronic disease has broad implications for society, with large potential benefits from improved health and productivity.

## 1. Introduction

Common chronic conditions include cardiovascular and cerebrovascular disease, cancer, diabetes, metabolic syndrome, and obesity, neurocognitive disorders, and immune dysfunction such as autoimmune disease. These are leading causes of morbidity and mortality in developed countries, and are increasingly prevalent in developing nations [1–3].

While average life spans lengthened through recent history, rising rates of noncommunicable chronic diseases in younger people mean that escalating numbers are spending an increasing proportion of their life coping with sickness, rather than enjoying health [4]. Indeed, chronic diseases associated with obesity may even turn the tide of improvements in average lifespans [5], previously gained from diverse advancements in public health and medicine such as in maternal and neonatal care and improvements in management of infectious diseases, trauma, and cardiovascular events. 

Chronic disease is crippling some economies as countries struggle to develop [3] in the face of rising healthcare costs, pervasive individual suffering, beleaguered families caring for afflicted loved ones, and truncated opportunities as workers fall ill during what should be their most productive years. 

It was recently conservatively estimated that costs in the United States of environmental disease in children alone amounted to a staggering $76.6 billion in 2008, just from “lead poisoning, prenatal methylmercury exposure, childhood cancer, asthma, intellectual disability, autism, and attention deficit hyperactivity disorder” [6]. The wide-spread implications are vividly illustrated by considering neurocognitive disorders, with a small IQ decrement across society. As abilities and intellect are reduced among the best and brightest, we lose potential leaders and innovators, while simultaneously costs mount for continuing care needed by larger numbers at the bottom end of the IQ and abilities spectrum [7].

Searching for reasons for increased chronic diseases in the young, their ailments cannot be ascribed to reduced mortality from infectious disease. Similarly, genetics may predispose individuals to chronic disease, but this cannot account for rapidly increasing prevalence within a generation or two. This leaves us with pervasive environmental factors [1, 3]. While research centers and international health organizations devote considerable time and attention to the issue of toxicant exposure and bioaccumulation of xenobiotics within the human body [8], direct connections to prevalent chronic disease are rarely made, and initiatives to tackle chronic disease may not even mention environmental or occupational exposures to toxicants, as was the case for a 2010 report prepared for the World Health Organization, “Tackling Chronic Disease in Europe” [9]. 

Other prominent authorities such as the U.S. President's Cancer Panel decry that chemical assessment, regulation, and enforcement are woefully inadequate to protect public health and that environmental and occupational exposures are rarely suspected or queried in the differential diagnosis of disease [10]. 

In this paper we provide a brief overview of toxicants and associated mechanisms that may be contributing to chronic disease, discuss how links between toxic exposures and adverse outcomes are explored and regulated, and outline measures that may be adopted by health care practitioners and individuals to improve health. These measures include reducing exposures, counteracting effects, and enhancing metabolism and excretion of toxicants.

## 2. Causes of Chronic Disease

Some chronic diseases may be initiated in susceptible individuals by single “germs,” such as acquired immune deficiency syndrome (AIDS) from human immune deficiency virus (HIV), ulcers from *Helicobacter pylori,* or Lyme disease from *Borrelia burgdorferi*, but most major chronic diseases do not have a single cause.

Modifiable factors such as smoking, alcohol, lifestyle (e.g., exercise), and nutrition top lists of contributors to cardiovascular disease, cancer, and obesity/metabolic syndrome/diabetes [1]. The view is weakening that genetic makeup predestines one to disease, as understanding grows of potential remediation of biochemical abnormalities underlying genetic predisposition to ill health [11]. Indeed, a small, shrinking fraction of chronic disease is attributed directly to genetic makeup, as recently discussed for cancer [10] and autism [12]. Instead, gene-environment interactions are very plausibly postulated, such as Apolipoprotein-E 4 interactions with heavy metals associated with Alzheimer's disease [13] or expression of glutathione genes and mercury body burden [14]. Such observations led to development of the field of toxicogenomics, with Judith Stern of the University of California at Davis stating, “genetics loads the gun, but environment pulls the trigger” [15]. In this context, George Church of Harvard Medical School commented that the conversation has evolved from, “Here is your destiny, get used to it!” to “Here is your destiny, and you can do something about it!” [16].

The related field of epigenetics is also coming into focus. Environmental factors ranging from stress to various chemicals may alter methylation of DNA, which in turn modifies DNA expression. Without altering the genetic code, individuals' morphology and predisposition to chronic disease are affected by exposures early in life or even exposures in previous generations, predating conception [17–19].

### 2.1. Environmental Contributors to Chronic Disease

In a recent, extensive review of associations between early exposures to various chemicals and chronic disease throughout life, Cooper et al. highlight the inadequacy of current scientific methods to tackle the causal puzzle for multiple related conditions (e.g., obesity, metabolic syndrome, cardiovascular disease, type 2 diabetes, and Alzheimer's and Parkinson's diseases), that may be associated with lower socioeconomic circumstances (with poorer diet and housing and increased stress) as well as with multiple potential contributory environmental factors (e.g., air pollution, heavy metals, and various endocrine disrupting chemicals) [20]. The importance of sowing the seeds of health early in life prompted Leiss and Kotch to issue a recent “wake-up call” regarding the importance of environmental health for mothers and children, to tackle intensive and extensive exposures involved in gene-environment interactions, impairing development in a multitude of ways [21]. They call for improved environmental regulation and control, better public education to combat avoidable exposures that are routinely occurring as a result of ignorance, more research, environmental justice, and coverage of environmental health in training of health care professionals.

With the caveat of noted limitations of reductionist approaches in examination of environmental causes of chronic disease, we summarize effects of identifiable groups of chemicals. The point of this summary is neither to depict the weight of evidence nor to be completely comprehensive but to illustrate the multiplicity and complexity of relationships between chemical exposures and health.

#### 2.1.1. Toxic Elements

These elements such as arsenic [22], cadmium [23], lead [24], and mercury [25] are typically found in drinking water, foods, dust, fish, dental amalgams, consumer products, and old pesticides. They are probable or established carcinogens that bind with sulphydryl groups in proteins and inhibit enzyme function, accumulating in organs such as the brain, kidney, liver, and bone, where they cause neurological dysfunction, organ compromise, and skeletal fragility. Among other wide-ranging effects of these chemicals are endocrine disruption by lead and immune system impairment by mercury. There are many other naturally occurring elements of concern, including fluoride, aluminum, and uranium [26].

#### 2.1.2. Naturally Occurring Substances

These include molds and their volatile metabolites, as well as animal, plant, and food allergens. In this context, it is increasingly recognized that sensitization to a variety of substances does not necessarily involve classic acute reactions, but that sensitivities to diverse substances, including foods, may be playing a role in diseases such as autism [27]. In parallel, sensitivities may arise to diverse man-made chemicals, not commonly thought to be associated with naturally occurring environmental factors [28–30].

#### 2.1.3. Pesticides

These (e.g., insecticides, herbicides, and fungicides) have diverse modes of action to kill, repel, or otherwise control pests (e.g., insects, weeds, and rot fungi). Pesticides are the only chemicals manufactured and spread in the environment specifically to be toxic, with a tiny fraction of broadcast/sprayed chemicals reaching their targets. In her famous book *Silent Spring*, Rachel Carson detailed ecosystem effects of pesticides following World War II, and subsequently poisoning of populations and military with herbicides used in the Vietnam War catapulted pesticides into the public eye. Some persistent pesticides now banned in developed countries continue to be used in underdeveloped countries, and residues are still in the environment and have accumulated towards the poles. Various present-day pesticides have been linked to cancers, and neurological, endocrine, developmental, reproductive, respiratory, and immunological disorders [31–33]. Pesticides produced in tissues of genetically modified crops, and also applied in large quantities to exploit crops' resistance to herbicides, were recently found in women and cord blood [34]. A popular means to reduce risk is to reserve most pesticides for the most essential uses, but to use least-toxic options for landscaping, in order to minimize children's exposure [35].

#### 2.1.4. Persistent Organic Pollutants (POPs)

These are a large group of diverse chemicals defined by their longevity in the environment and in the body. They include dioxins and furans (herbicide contaminants, incineration products, environmental product of antimicrobial “triclosan”), polychlorinated biphenyls and polybrominated diphenyl ethers (old and newer flame retardants), polyfluorinated stain repellents, antiwrinkle and nonstick compounds (e.g., Teflon), and organochlorine chemicals (old pesticides—e.g., hexachlorobenzene, DDT, and metabolite DDE). POPs generally have low solubilities in water and are lipophilic (or in the case of some polyfluorinated compounds are surfactants, concentrating at phase interfaces). POPs biomagnify up the food chain as they accumulate in adipose tissue, where they may represent a pool of toxicants with diverse health effects including carcinogenesis [36]. Many POPs with conjugated ring structures are endocrine disruptors as they interact with binding sites for hormones. POPs may also bind with the aryl hydrocarbon receptor (AhR), which elicits a cascade of “dioxin-like” toxic responses [37]. Knowledge of AhR binding toxicities led to regulation of chemicals on this basis, but “nondioxin-like” POPs are also linked to health outcomes such as diabetes [38]. Diverse POPs now present a complex picture in the context of various pervasive health conditions such as obesity, metabolic syndrome, diabetes, and endometriosis [39–45].

#### 2.1.5. Volatile Organic Compounds (VOCs)

These are another large group of chemicals, defined by their lower molecular weight and volatility [46]. They include solvents, fuels such as gasoline, and fragrance ingredients [46, 47]. They may interfere with cellular membranes and cause diverse neurological effects. Some such as formaldehyde, benzene, and synthetic musks are carcinogens, and some fragrance ingredients are also known sensitizers [29, 30, 48, 49].

#### 2.1.6. Plastics

These are manufactured from monomers, that are chemically strung together to form polymers. Toxic concerns regarding various plastic vary widely, with vinyl standing out for toxicity of the monomer and additives, as well as challenges with disposal [50]. Unless plastic is degraded (with heat, ultraviolet radiation or chemical attack, such as the xenoestrogen bisphenol-A released from polycarbonate or epoxy liners in food containers), it is generally inert, but chemicals dissolved in the plastic may leach out. “Plasticizers” to improve flexibility or resiliency include endocrine disrupting phthalates, and stabilizers and dyes may contain toxic metals such as lead or cadmium (leading to recalls of children's items such as toys, etc.).

### 2.2. Mechanisms of Toxicity in Chronic Diseases

The web of effects and interactions with diverse toxicants affecting multiple metabolic and physiological pathways may seem to defy reductionist approaches to single-chemical toxicities and causation of conditions. Nevertheless, with large population studies and sophisticated analyses, effects of toxicants and interactions with toxic as well as beneficial chemicals/nutrients may be discerned. First, some common biochemical effects merit highlighting.

#### 2.2.1. Oxidative Stress

This features in development and exacerbation of many chronic conditions [29, 51, 52], such as allergy and autoimmunity [53, 54], cancer [55], cardiovascular disease [56–58], diabetes [59], neurological compromise [60], lung disease, and sensitization and pain syndromes [28, 61]. Mitochondria are a particular focus of these effects [62].

#### 2.2.2. Endocrine Disruption

This is apparent in altered puberty and sexual development as well as energy utilization, glucose sensitivity, and neurological development. Indeed, interrelated effects on insulin signalling, oxidative stress and vascular health have prompted Alzheimer's disease to be dubbed “diabetes of the brain” [63, 64].

#### 2.2.3. Genotoxicity

This has been studied extensively for single chemicals but is now recognized as only one aspect of development of clinically relevant cancers. Immune surveillance, oxidative stress, and stimulation of growth by infections, inflammation or endocrine disruption may also contribute to carcinogenesis or mutagenesis. More common than changes to DNA sequence are epigenetic changes that alter expression of the genome, and thereby induce a disease state [18, 65].

#### 2.2.4. Enzyme Inhibition

This is a direct effect of pesticides designed to bind with receptors, or of toxic metals that bind with protein sulfhydryl groups, thereby inactivating a wide range of enzymes, with diverse adverse effects. For example, altered porphyrin profiles may be detected with metal toxicity due to enzyme inhibition [26].

#### 2.2.5. Dysbiosis

Dysbiosis or disruption of the human microbiome has become an area of intense research and clinical interest with the recently initiated Human Microbiome Project by the US National Institutes of Health (http://commonfund.nih.gov/Hmp/). Infection and various toxicants including some heavy metals have the potential to alter gut flora, and thus modify various functions of the gastrointestinal tract including digestion, bioavailability/absorption, elimination, detoxification, and immune function [27, 66–69]. Conversely, gut microbes may transform toxicants such as arsenic or polyaromatic hydrocarbons, altering toxic effects [70, 71]. These phenomena elicit diverse health sequelae for the gastrointestinal tract as well as diverse systemic toxicities such as inflammation and neurological effects [72–74].

Other toxicant-related mechanisms of harm are increasingly being recognized, including immune dysregulation [29], autonomic nervous system impairment [75], biochemical binding (e.g., carbon monoxide supplanting oxygen on hemoglobin, or heavy metals supplanting zinc on glutathione), and overall a toxicant induced loss of tolerance (TILT) [30].

## 3. Major Chronic Diseases

### 3.1. Obesity/Metabolic Syndrome/Diabetes

These are linked in complex ways to diverse POPs, including polychlorinated biphenyls, dioxins and flame retardants [76]. These chemicals may interfere with thyroid function, and as xenobiotics stored in adipose tissue are released into the blood stream during weight loss, they may undermine efforts to lose further weight [77]. Other toxicants such as arsenic or cadmium that increase oxidative stress in the pancreas may also contribute to diabetes.

### 3.2. Vascular Disease

This manifests in cardiac, renal, cerebral and peripheral disorders. Strong connections with toxic metals have been established, with oxidative stress implicated as a central mechanism. Chelation of toxic elements such as lead may decrease renal impairment [78]. Unfortunately, a large chelation trial for vascular disease currently under way is not measuring toxic elements [79].

### 3.3. Cancer

This is an extensively studied endpoint, with many carcinogens identified in occupational settings. Carcinogenicity of lower exposures and with mixtures are sometimes less clearly defined, as discussed above. Nevertheless, there is consensus that there is no “risk-free” or threshold dose of genotoxic substances. Environmental chemicals may contribute to cancer by altering DNA or its expression, by stimulating rapid growth and confounding cell repair mechanisms in hormone-sensitive tissues or via inflammation, or by impairing immune surveillance.

### 3.4. Neurocognitive Impairment

This, including reduced IQ and aberrant behaviour, is linked to early life exposure to a wide range of environmental toxicants, including heavy metals, various POPs and pesticides [80]. Effects may be immediate (e.g., problems with learning, attention and aggression) or delayed (e.g., increased predisposition to Alzheimer's or Parkinson's disease). A few common mechanisms include endocrine disruption (e.g., polyhalogenated biphenyls mimic thyroid hormone), direct inhibition of neuronal growth by toxic metals (e.g., mercury), or interference with signal transmission by pesticides or toxic metals.

### 3.5. Multi-System Complaints

Patients significantly disabled with multisystem complaints are increasingly commonly presenting themselves to clinicians. Recent evidence suggests that TILT is a pervasive mechanism of illness involving several organ systems concurrently [29, 30].

## 4. Timing and Vulnerabilities

The exquisite vulnerability of the young and unborn was tragically clear when mothers in Minimata, Japan, who were coping with relatively mild symptoms from methylmercury in the fish they ate, gave birth to children with severe neurological damage [81]. Unique vulnerabilities of the fetus and child arise when chemicals interfere with windows of differentiation of tissues *in utero* and during development. Lead, pesticides, and other chemicals at doses that do not overtly impair the mother may harm her offspring, with immediately evident or delayed neurological, endocrine or other effects [82–85]. Indeed, the mother may impart bioaccumulated toxicants (e.g., lead or cadmium from her bones, or lipophilic pollutants from her adipose tissues) to her fetus through the placenta, or infant via breast milk. Nevertheless, breast milk is undoubtedly the best food for infants.

Duration of exposure is another aspect of timing. Haber's Rule and related mathematical treatments of toxicity model that when a toxicant is persistent (or quasipersistent with ongoing exposure to a ubiquitous toxicant such as bisphenol-A), or the chemical's effect is irreversible, then either a low, chronic dose or a high acute dose may both result in toxic effects [86]. For many toxicants, there is no “nontoxic” dose. The public health and regulatory response to this situation is often to strive for levels that are “as low as reasonably achievable,” typically accompanied by arguments with commercial enterprises over the “reasonableness” of costs of remediation, abatement, and alternative processes and products.

## 5. Exposure and Dose

In common usage, “exposure” is sometimes equated with dosage, with toxicant levels in the environment, drinking water, foods and products subject to regulation and public health measures. Nevertheless, toxicologists and health care professionals parse exposure and doses. For instance, the presence of a chemical in the environment (e.g., house dust) does not necessarily mean that a child will crawl in it and get it on his fingers. Even if he licks his fingers and ingests the myriad chemicals in house dust, intestinal absorption varies according to the chemical, age and state of the child's development, nutritional status, and other materials and foods in the intestine at the same time. The dosage to a target organ such as the brain will once again be modified by absorption into cells and across membranes, and by metabolism and excretion. Measurement of a toxicant in most target organs or tissues is not feasible in living human study participants, so surrogate levels may be measured in blood, urine, hair, sweat, feces, and even meconium, cord blood, nails and deciduous teeth [82, 87–90].

## 6. Responses to Toxicants

### 6.1. Recognition

A potential risk must be recognized before any response is possible. This includes the classic questions: what? (is the substance), where? (is a substance made/found), how? (does toxicity manifest), who? (may be affected), and why? (is it used, and are there alternatives that pose less risk).

Beyond prioritization and assessment of chemicals, measures to reduce environmental and individual exposures occupy considerable resources of government agencies and ministries, and international bodies such as the World Health Organization. Increasingly, emissions and product content data, as well as toxicology data necessary for chemical assessment and registration for use, are being required from industries, manufacturers and importers.

### 6.2. Chemical Assessment

Numerous, diverse environmental exposures merit scrutiny for health effects, including factors affecting chronic disease. As well as age-old toxic elements (mercury, lead, cadmium, arsenic, etc.), and allergens and organic chemicals such as mold metabolites, we and our off-spring carry many chemicals in our bodies that mankind has only recently encountered [8]. Approximately 80,000 novel chemicals have been registered for use with the United States' Environmental Protection Agency since World War II, but the vast majority have not been assessed for human health or environmental effects [91].

Around the world, various approaches are being used to address the conundrums posed by such vast numbers of novel chemicals. Environment and health ministries and regulatory agencies in North America have for years been prioritizing lists of chemicals including pesticides, assessing the most urgent ones, and restricting uses or banning occasional chemicals for particular uses. Europe instituted the Registration, Evaluation, Authorisation and Restriction of Chemicals (REACH) regulation in 2007, administered by the European Chemicals Agency (ECHA), placing greater responsibility on industry to provide data and to manage risk [92].

Risk is typically initially screened on the basis of inherent toxicity (often acute), whether the chemical is persistent or bioaccumulative, and the potential for exposure. Toxicological risk assessment is carried out by chemical agencies, on the basis of animal toxicology research supplied by industrial applicants. Typically a dose level is determined that produces no (serious) observable adverse effect in an animal, which is then extrapolated to a lower exposure (e.g., level in air, water, food, or a product, or an application rate for a pesticide) that is said to pose a reasonable risk to humans. As long as the permissible exposure is well above levels observed in the real world, then the chemical or product may be registered for sale and use. This paradigm does not address multiple exposures with potential cumulative or synergistic effects on a particular system such as the nervous system [93], although efforts are evolving to assess small groups of chemicals commonly found together [94], and to carry out *in vitro* high throughput, rapid biochemical and cell system screening, *in lieu* of animal testing [95].

The catch with toxicological testing is that it would be illogical for an industry to conduct testing at environmentally relevant doses, because the regulatory framework means that observations of effects will preclude registration. A long-standing tenant of toxicology, that all effects at low doses are presaged by effects at higher doses, is captured as “The dose makes the poison,” a common paraphrase of Paracelsus' writings from the 16th century. Research now contradicts this view that toxicity becomes apparent at higher doses and is not detectable below a certain level, as non-monotonic dose response curves are well recognized to be usually associated with endocrine effects [96]. Indeed, the American Chemical Society, with extensive membership among the chemical industry, recognizes that low-dose, endocrine disrupting effects are scientifically undermining the toxicological testing that underpins current chemical regulation [97].

Another obvious shortcoming of regulatory animal toxicology is that humans are not rodents. Human studies of toxicants are ethically intransigent, and thus necessarily subject to limitations of observational studies, but health researchers and professionals argue persuasively why and how epidemiology could and should be given greater weight in chemical assessment [98].

Faced with fundamental doubts overshadowing today's environmental standards and regulatory decisions, some countries such as Sweden now consider inherent safety, how essential a chemical or product is, and whether a less toxic, lower risk substitute is feasible; this is the Substitution Principle, a means to put into operation the Precautionary Principle [99]. The Substitution Principle is in part a pragmatic response to the enormity of the numbers and diversity of anthropogenic chemicals, as well as the logistical impossibility of scientifically assessing all chemicals, let alone combinations [99]. In essence, the least-toxic, and most environmentally sustainable options for a particular product or application are the ones that are permitted. Inclusion of the null option—that a particular product is not necessary for society—allows regulators to rule out a number of chemicals from those needing to be further assessed while providing a strong incentive for “green” chemistry.

## 7. Public Health

With rules laid out as to allowable uses and levels of toxicants, protection of public health falls into the purview of a variety of professionals, from environment, agriculture, natural resource, and health ministries to local public health.

### 7.1. Monitoring and Detection

Pollution and human exposure may be tracked on many levels, by governments monitoring and reporting toxicants from large scale industrial emissions; environmental levels in air, water, soil, and wildlife; individual exposures in foods, drinking water and consumer products; and levels in people themselves with population surveillance. Publicly available data may serve many purposes, from deciding to avoid vigorous exercise on the smoggiest days, to investigating contaminated sites and pollution sources in one's neighbourhood. Governments regularly publish online data such as daily air quality, and may announce product recalls (e.g., children's products with lead or cadmium, or baby formula with melamine). A list of some helpful websites addressing chemical assessment and monitoring is provided in Table 1 (many additional sites are available for individual countries). Nongovernmental organizations also carry on myriad public education campaigns such as recommending foods with lower levels of pesticides [100] or personal care products with lower levels of chemicals of concern [101], or even identifying houses at risk for child lead exposures [102].

Governmental regulation provides a bottom line for use of and exposure to potential toxicants, but chemicals have historically been assumed “innocent until proven guilty,” and there is a long history of examples such as pesticides being banned after decades of use, and uses and limits for chemicals such as lead falling as scientific knowledge improves. Evolution of public health initiatives as risks are recognized may drive innovation; for instance, more stringent standards are possible as water and waste treatment technologies improve, and best practices for pest management change when the array of permitted chemicals is limited.

Individual choices (e.g., when shopping) will be affected by education and perception regarding risk, as well as the cost, feasibility, and identification (with honest, meaningful labelling) of alternatives. Medical practitioners themselves should be knowledgeable, and have the resources to educate and facilitate their patients making the best choices for their personal, family, patient and community health. On a broader scale, the voice of the medical community has excellent credibility in setting public policy to promote health.

## 8. Clinical Considerations Regarding Toxicants

The reality of the contemporary world is that toxicants are ubiquitous and, while avoidance is central to any management strategy, toxicants are not entirely avoidable. This has always been true for naturally occurring elements, but the recent onslaught of anthropogenic substances poses new challenges for an individual's biochemistry and hence for the clinician.

Although identifying the epidemiologic patterns of toxicant exposure and health sequelae has been one focus of attention, some within the clinical world are asking, “So what do we do about it?” The realization that many throughout the world already possess a significant body burden of anthropogenic compounds [8, 103], juxtaposed with the emerging reality of serious potential health sequelae associated with toxicant accrual has inevitably led some scientists and clinicians to consider possible measures to reduce the toxicant burden in the human body in order to reduce the risks associated with accretion of diverse compounds.

Some toxicologists and clinicians, often untrained in environmental health sciences, consider strategies to eliminate toxicants from the body to be myths lacking in merit, assuming that all toxicants are metabolized and excreted naturally and concluding that “there's nothing that does anything to hasten the detoxification process” [104]. Emerging evidence, however, challenges this misconception. The contention that the body has an inherent ability to eliminate quickly all adverse chemical compounds is inaccurate, as many toxicants with long half-lives accrue in tissues or blood, thus maintaining long-term potential to inflict damage. Metals such as lead and cadmium, and many halogenated compounds (e.g., flame retardants, nonstick compounds, stain repellents, and organochlorine pesticides), are persistent human pollutants with extended half-lives. Unfolding research reveals ineluctable evidence that various interventions facilitate elimination of retained compounds [87, 105], with the objective of diminishing the risks associated with biologically stockpiled poisons. Although more extensive reviews of various modalities to eliminate toxicants can be found in other works [105], we present an overview of potential approaches that can be employed to facilitate the removal of accrued toxicants.

With a wide range of distinct chemical compounds, each with a unique chemical structure and a potentially distinct way of interfacing with human biochemistry, there is no single mechanism or pathway for the body to eliminate the whole spectrum of 21st century chemical toxicants. Thus, when attempting to detoxify the human body, it is first important to explore the specific accrued toxicants comprising the total chemical burden, and to employ effective methods to facilitate excretion of various components.

### 8.1. Determination of Body Burden of Toxicants

When a patient with evidence of potential toxicant-related health problems presents to a clinician trained in environmental health sciences, an attempt is generally made to identify which adverse chemicals are retained within the body, in order to employ specific interventions to address each of these compounds. With the vast array of toxicants that individuals are exposed to, how does one comprehensively determine which toxicants are present and then assess the extent of the total body burden?

As clinical laboratory methods to assess many toxicants are not extensively validated with meaningful reference ranges, there is limited ability at this time to investigate a broad range of specific chemicals. Blood and urine are most commonly sampled to assess levels of retained toxicants. Apart from difficulties interpreting results in the absence of population-specific reference-limits, these measurements may be significantly flawed as indicators of bioaccumulation because many compounds sequester in tissues; they do not remain in blood and may not be readily excreted in urine. Thus testing of whole blood or serum generally does not adequately detect toxicants that are being stored primarily in organs, bone, muscle or adipose tissues [26, 87]. As well, levels of toxicant compounds in blood and urine can also fluctuate rapidly as a result of nutrient or pharmaceutical use, caloric restriction, hydration, underlying nutrient status, thermal changes, or exercise [106–110].

For clinical purposes therefore, blood or urine testing to determine the total body load of toxicants may underestimate the level of accrual for many toxicants. Testing of other tissues and bodily excretions has also been explored, including salivary testing, hair analysis, stool sampling, perspiration testing, breath analysis, provocation testing, as well as biopsies of fat tissue. Recent evidence confirms, however, that there are limitations with each of these approaches. Hair samples, for example may only reflect selected toxicant levels in the blood stream for the last few weeks, while stool samples only assess what is being eliminated through the gastrointestinal tract. Fat biopsy research confirms that toxicants sequester differently within different fat compartments, with toxicant concentrations varying widely among adipose tissue sites [111]. Although selected testing techniques for some toxicants can be helpful as an indication of toxicant bioaccumulation, attempts to accurately and comprehensively delineate the accrued level of each toxicant compound are impractical clinically and prohibitively expensive. The results are imprecise at best, and are prone to false negatives in the sense that a low blood value, for instance, may simply not reflect high levels in the bone, or a vital organ such as the kidney or brain. Values in urine, hair and feces by definition reflect the ability to excrete rather than the body burden of a toxicant, and impaired excretion may result in greater accretion and potential adverse effects, leading to the paradoxical finding that body burden is apparently lower in a population whose health is in fact being affected by a toxin, as was seen in children with autism [112–114].

So what is a reasonable clinical approach to patients who appear to have been harmed by bioaccumulative toxicant exposures?

### 8.2. Pragmatic Clinical Management

With currently available knowledge and technologies, three fundamental clinical steps should be considered in the initial assessment and patient care planning for those with potential toxicant-related health problems:

- perform a comprehensive history to endeavour to identify past and present exposures [115],
- order selected toxicant testing as is feasible in each situation,
- use clinical judgement to institute low-risk elimination strategies directed towards the individual patient.

Rather than specialized treatments for each specific toxicant identified, in this paper, we present a general approach to detoxification. This is an ongoing area of research, and other works are available that provide further particulars in the management of specific toxicant categories and individual chemical exposures [87, 88, 105].

The clinical approach to human elimination of accrued compounds generally involves three successive stages:

1. personal avoidance,
2. securing efficacy of endogenous mechanisms for toxicant elimination,
3. directed interventions to facilitate removal of accrued toxicants.

#### 8.2.1. Avoidance

It is sometimes bantered about in environmental health science circles that the three most important principles in addressing the problem of widespread toxicant exposure are “avoid, avoid, and avoid.” As previously discussed, through means of personal education, notification (e.g., labelling) and actions (e.g., remediation of living spaces and changes in diet) as well as governmental and industrial regulation with enforcement, minimization of further exposure is achieved. This is fundamental to any successful strategy to diminish the toxicant burden of individuals and populations.

From a clinical perspective, it is useful for the health provider to perform a detailed inventory of potential exposures. By means of a meticulous environmental health questionnaire [115], most common exposures can be identified. An inventory of the six routes of possible sources of exposure should be undertaken:

- ingestion,
- breathing,
- skin contact,
- olfactory transmission via smell,
- vertical transmission (i.e., mother to fetus/infant),
- penetration of body tissues through processes such as surgery, dentistry, injection, or vector routes.

By identifying exposures and apprising individual patients regarding where and how they are being contaminated, patients are empowered to avoid further chemical contamination. With ongoing exposures minimized, the human organism is able to devote resources and energies of detoxification physiology to metabolizing and excreting retained compounds, with less devoted to ongoing exposures.

#### 8.2.2. Securing Efficacy of Endogenous Mechanisms for Toxicant Elimination

The human body has enormous potential to detoxify foreign compounds through various physiological mechanisms. Endogenous detoxification of metabolic waste products as well as foreign toxicants is a primary physiological function, that requires considerable energy. Major organs of detoxification include the liver, kidney, skin, and lungs. When toxicants are identified by physiological processes within the body, pathways of excretion are mobilized to diminish toxicity and to eliminate the xenobiotic compounds. The particular pathways used to excrete specific substances will depend on the chemical properties of the particular agent in question. Potential pathways include metabolism or conjugation to form water-soluble compounds for renal excretion, metabolism to less toxic forms (e.g., methylation of arsenic), conjugation with biochemicals such as glutathione for gastrointestinal elimination or intracellular metallothionein binding of heavy metals [26].

The ability to eliminate undesired compounds, however, depends completely on the physiological functioning and biochemical status of the individual. Anything that impairs full functioning of detoxification biochemistry, such as nutritional deficiencies, will preclude proper elimination of toxic substances. Accordingly, it is imperative that health providers understand the fundamentals of detoxification physiology and biochemistry to secure functioning of the organs of elimination [26].

Clinical history and physical examination can provide clues to the status of physiological function and potential causes for impairment. A history of substance use, for example alcohol or medications known to be hepatotoxic, can be helpful in assessing liver function. Nutritional biochemistry testing, urinary organic acid testing, and biochemical markers for function of organs such as liver and kidney, can be employed to assess physiological status and function. Any impediment to proper physiological functioning should be addressed.

Remediation of disordered nutritional biochemistry is a fundamental component of patient care. For example, the protein molecule glutathione is a prerequisite component of cellular detoxification as well as an essential pillar in hepatic conjugation biochemistry. Individuals with ongoing exposures or toxicant bioaccumulation often have diminished stores of glutathione, and thus require ongoing repletion. Optimal nutrition through dietary instruction, correction of disordered biochemistry and physiology, and use of directed supplementation as dictated by laboratory testing is required for efficient physiological functioning of elimination pathways.

It has also been observed that despite biochemical competency, the human organism is not able to excrete some chemical toxicants effectively. A major reason for the failure of some compounds to be eradicated effectively is because of recycling within the body through reabsorption in the enterohepatic circulation [116] or reuptake in kidney tubules [117]. Accordingly, some toxicants are conjugated and released from tissues into the bloodstream for excretion, but are then reabsorbed back into the body. Additionally, some retained compounds deposit in specific tissues such as bone, fat, and muscle, where they will bioaccumulate and alter physiological functioning within these tissues. Some compounds will also remain in blood to some degree, frequently bound to plasma proteins [88]. Interventions to enhance excretion of retained compounds can be invaluable in diminishing morbidity associated with toxicant accrual.

Environmental determinants such as toxicant bioaccumulation in some situations may interfere with normal physiological function and thus necessitate intervention. For example, vitamin D (an essential biochemical that regulates genetic expression, and facilitates absorption of calcium from the gut) may potentiate absorption of toxic elements such as lead, aluminum, and cadmium, that in turn may impair metabolism of vitamin D [118]. Mercury contamination may alter gastrointestinal absorption of required nutrients resulting in deficiencies and thus precluding normal physiology. Some persistent organic pollutants released from adipose tissue during weight loss, caloric restriction or exercise may suppress thyroid function [77]. Adverse effects from toxicant bioaccumulation may cascade within the body—some toxicants, for example, may alter immune function, which may in turn spawn autoimmunity [119] and thus engender abnormal physiological functioning. In difficult cases, consultation with experienced environmental health specialists may be required.

#### 8.2.3. Directed Interventions to Facilitate Removal of Accrued Toxicants

Empirical research has shown that various strategies can be employed to assist with the effective removal of some accrued toxicants. Research into such strategies, however, remains at an early stage in the continuum of clinical science as the problem of widespread toxicant bioaccumulation is a newly recognized phenomenon for clinical medicine. Accordingly, adequate evidence-based research to objectively confirm or refute the alleged efficacy of assorted “detox strategies” is often lacking. A few strategies and a general approach to clinical detoxification are highlighted here for consideration. A more detailed discussion of commonly employed strategies to facilitate detoxification can be found elsewhere [105].

#### 8.2.4. Thermal Depuration (Sweating)

The skin is a major organ of detoxification, and a vast array of toxicants are able to be excreted to differing degrees via perspiration [87, 105, 120]. Various researchers and clinicians have endeavoured to take advantage of this dermal mechanism to facilitate excretion of accrued compounds and toxicological biomonitoring has confirmed that body burdens of many toxicants diminish with therapy to induce sweating [105, 121, 122]. Some chemical agents such as perfluorinated compounds, however, do not seem to be readily excreted [88]. Despite much attention given to saunas with heaters emitting at specific electromagnetic frequencies, research to date suggests that there is no difference in toxicant excretion rates between perspiration that occurs through infrared sauna, dry or wet regular saunas, or exercise [87, 123].

#### 8.2.5. Selected Medications

These perform a useful role in facilitating the elimination of some compounds. For example, judicious use of chelators, or agents which strongly bind to some toxic elements have been demonstrated to assist in the removal of such toxicants [124, 125]. Chelating agents such as dimercaptosuccinic acid (DMSA) are generally safe and effectively bind to metals such as lead and mercury to enhance excretion rates and to prevent enterohepatic reabsorption of these compounds [125]. Marked clinical improvement has been noted in metal contaminated patients who have been treated with use of such medications [126]. The use of concomitant strategies, such as foods and supplements to increase glutathione, to enhance mobilization of toxicants from tissue storage sites may significantly increase the rate of elimination from the body when used along with chelators.

Bile acid sequestrants such as cholestyramine have recently garnered increasing attention as compounds that bind to some persistent compounds in the gastrointestinal tract to prevent enterohepatic reabsorption [88]. These agents may be useful with such persistent agents as perfluorinated compounds [88] and have been clinically effective in patients with mycotoxin accrual after mold exposure [127]. As medical interventions to enhance elimination of toxicants is a newer area of clinical research, much remains to be studied in order to develop evidence-based medication protocols for the removal of some toxic compounds.

#### 8.2.6. Selected Foods and Supplements

Direct evidence of specific benefits in humans of diet and dietary supplements is often of limited applicability beyond the subject population, because of interactions with factors such as regional environment (e.g., cadmium or selenium in the soil), lifestyle, dietary practices, levels of nutrients such as vitamin D from sun exposure, poverty, and alcohol and tobacco use. Some medical evidence exists, however, that nutritional status (e.g., calcium, zinc and iron repletion) modifies absorption of toxic elements such as lead, and that beneficial effects of omega-3 fatty acids and selenium in fish somewhat counteract toxicities of methylmercury and persistent organic pollutants [128, 129]. 

Research questions are often much more amenable to animal and tissue culture research, and this body of evidence has confirmed traditions that some foods and supplemental nutrients are enormously valuable in facilitating excretion and reducing biochemical toxicities of toxicants. Although by no means an exhaustive list, some supplements include curcumin in the spice turmeric [130], alliums [131], plant flavonoids such as quercetin [132], selenium [133], algal products *Parachlorella* [134, 135] and *Chlorella* [136, 137], naturally occurring organic acids [138], folate requisite minerals, and dietary fibre [139] as well as mixed antioxidants [140] appear to be of great value to reduce the damage associated with toxicant exposure. The mechanisms of action may include preventing absorption of toxicants, facilitating elimination of accrued toxic compounds, hindering enterohepatic recycling of some persistent compounds, and diminishing toxicity through protective mechanisms. Insoluble carbohydrate and other fibre consumed in the diet, for example, appears to act like a sponge and increases the removal of adverse agents such as mycotoxins and POPs, perhaps by diminishing reabsorption through the enterohepatic circulation, and thus increasing elimination.

One example is a supplemental product called *Chlorella*, an algae from the sea, that has recently garnered much research attention for its unique properties in facilitating detoxification and preventing absorption of adverse compounds [134–137]. Recent research papers reported animal results where *Chlorella* appears to induce the excretion of mercury [135] and lead [134]. Ongoing study continues to elucidate the range of compounds that are bound and removed with *Chlorella* as well as with other assorted foods and supplemental nutrients. A caution, however, is that supplements may potentially contain toxicants that accrued as they grew, such as *Chlorella *and other biosorbents that are also noted for their ability to sequester toxic elements from their environment [141], fish oil that may contain POPs [142], or other products that may be contaminated for other reasons [143].

#### 8.2.7. Other Detoxification Modalities

With increasing recognition of ubiquitous toxicant exposure and bioaccumulation [8], a plethora of treatments called “cleanses” have become commonplace. Furthermore, “detox” clinics have sprung up throughout the western world allegedly to help individuals rid their bodies of accumulated toxicants. Unfortunately, there is limited research on many of the programs and therapies that are commonly used and scientific evidence is often lacking to support the audacious claims frequently made to vulnerable, sick people. While some interventions such as exercise [144, 145], induced sweating [87, 120], selected medications [124, 146] (including in those children with autism who have elevated levels of heavy metals [113, 147]), plasmapheresis [148, 149], some foods and some supplements [137, 138, 150], and other modalities show credible evidence of efficacy in removing toxicants, much research remains to be done in order to yield consistent evidential support for the increasing plethora of “detox” interventions. Using data available to date, a basic approach that can be used clinically, that incorporates the three successive steps for detoxification (avoidance, support of endogenous detoxification, and directed interventions) is presented for consideration.

## 9. Seven Steps General Clinical Approach to Detoxification

1. A detailed environmental history and exposure inventory, followed by patient education to effect adequate avoidance [115].
2. Specific toxicological testing if indicated according to the patient history [151].
3. Remediation of abnormal biochemistry, as identified by laboratory investigations [26].
4. A combination of optimal diet and supplemental nutrients may be utilized to secure adequate nutrition and sufficient biochemical reserve for detoxification [152]. Some examples include (this is not a comprehensive list) the following:
  - glutathione [153] and sulfur-containing foods (e.g., eggs, brassicas and alliums) and supplements such as N-acetylcysteine, taurine or methylsulfonylmethane to support glutathione and metallothionein synthesis,
  - folate and related nutrients (e.g., for arsenic metabolism and excretion [154]),
  - lipoic acid therapy may assist in detoxification of some compounds including mycotoxins resulting from exposure to certain molds [150],
  - adequate minerals such as calcium, iron and zinc, to reduce absorption of heavy metals [128],
  - *Chlorella* to decrease absorption and enhance excretion of toxicants is generally well tolerated [134–136, 155],
  - fiber to reduce absorption and facilitate elimination [139].
5. Regular sweating (with mineral repletion), with exercise or in a sauna, can facilitate transdermal excretion [87].
6. Daily exercise will enhance eliminate of some toxicants [144, 145].
7. Directed therapies for retained toxicants (as determined by laboratory investigations) may be implemented [88, 105].

### 9.1. In Summary

Health care professionals in government ministries, public health, research, and the clinic will only be successful against the onslaught of chronic, debilitating diseases once environmental contributors are recognized, researched and addressed. Clinical intervention to preclude further exposure and to detoxify the body of toxicants can be life changing for afflicted individuals [126]. In an epoch marred by the unleashing of numerous untested chemical toxicants, basic knowledge of environmental medicine should be provided in the training of all health care workers. History repeatedly demonstrates, however, that the translation of emerging scientific information with adoption of required clinical skills is usually not expeditious [156, 157]. Hopefully, in our modern era of rapid information transfer, the process of widespread problem recognition and solution implementation will be expedited to stem the tide of chronic disease that is said to be poised to bankrupt healthcare systems.

## Figures and Tables

**Table 1:** Selected websites for chemical information.

World Health Organization	http://www.who.int/
INCHEM (Chemical Safety Information from Intergovernmental Organizations)	http://www.inchem.org/
Concise International Chemical Assessment Documents (CICADs)	http://www.inchem.org/pages/cicads.html
Environmental Health Criteria (EHC) Monographs	http://www.inchem.org/pages/ehc.html
Health and Safety Guides (HSGs)	http://www.inchem.org/pages/hsg.html
International Agency for Research on Cancer (IARC)—Summaries and Evaluations	http://www.inchem.org/pages/iarc.html
International Chemical Safety Cards (ICSCs)	http://www.inchem.org/pages/icsc.html
IPCS/CEC Evaluation of Antidotes Series	http://www.inchem.org/pages/antidote.html
Joint Meeting on Pesticide Residues (JMPR)	http://www.inchem.org/pages/jmpr.html
KemI-Riskline	http://www.inchem.org/pages/kemi.html
Pesticide Documents (PDs)	http://www.inchem.org/pages/pds.html
Poisons Information Monographs (PIMs)	http://www.inchem.org/pages/pims.html
Screening Information Data Set (SIDS) for High Production Volume Chemicals	http://www.inchem.org/pages/sids.html
Centers for Disease Control and Prevention (USA)	http://www.cdc.gov/
Agency for Toxic Substances and Disease Registry (USA)	http://www.atsdr.cdc.gov/
National Institute for Occupational Safety and Health (USA)	http://www.cdc.gov/niosh/
Environmental Protection Agency (USA)	http://www.epa.gov/
National Institute for Environmental Health and Safety (USA)	http://niehs.nih.gov/
National Toxicology Program (USA)	http://ntp.niehs.nih.gov/
European Commission—Public Health	http://ec.europa.eu/health/index_en.htm/
European Commission—Scientific Committee on Emerging and Newly Identified Health Risks (SCENIHR)	http://ec.europa.eu/health/scientific_committees/emerging/index_ en.htm/
European Chemicals Agency	http://echa.europa.eu/
